# Assessing the genetic diversity of guava germplasm characterized by morpho-biochemical traits

**DOI:** 10.3389/fnut.2022.1017680

**Published:** 2022-09-30

**Authors:** Nayan Deepak Gangappa, Chandu Singh, Mahendra Kumar Verma, Madhubala Thakre, Amitha Mithra Sevanthi, Rakesh Singh, Manish Srivastav, K. Raghunandan, Chukkamettu Anusha, Vivek Yadav, Arumugam Nagaraja

**Affiliations:** ^1^Division of Fruits and Horticultural Technology, Indian Council of Agricultural Research, Indian Agricultural Research Institute, New Delhi, India; ^2^Division of Genetics, Indian Council of Agricultural Research, Indian Agricultural Research Institute, New Delhi, India; ^3^Indian Council of Agricultural Research, National Institute for Plant Biotechnology, New Delhi, India; ^4^Indian Council of Agricultural Research, National Bureau of Plant Genetic Resources, New Delhi, India; ^5^College of Horticulture, Northwest Agriculture & Forestry University, Xianyang, China

**Keywords:** qualitative, quantitative, germplasm, guava, variability, diversity

## Abstract

Amid environmental crises, a galloping population, and changing food habits, increasing fruit production with nutritional quality is a global challenge. To address this, there is a necessity to exploit the germplasm accessions in order to develop high-yielding varieties/hybrids with good adaptability and high quality fruit under changing environmental and biological conditions. In the study, a total of 33 morpho-biochemical traits enabled an assessment of the genetic variability, diversity, and structure in a collection of 28 diverse germplasm lines of guava. Results showed that highly significant genetic variability existed in the studied traits in the guava germplasm. The coefficient of variation values for the qualitative and quantitative traits varied from 23.5–72.36 to 1.39–58.62%, respectively. Germplasm Thai, Lucknow-49, Punjab Pink, *Psidium friedrichsthalianum*, and Shweta had the highest fruit weight (359.32 g), ascorbic acid content (197.27 mg/100 g fruit), total phenolic content (186.93 mg GAE/100 g), titratable acidity (0.69 percent), and antioxidant capacity (44.49 μmolTrolox/g), respectively. Fruit weight was positively correlated with ascorbic acid content; however, titratable acidity was negatively correlated with fruit weight. The principal component analysis (PCA) was 84.2% and 93.3% for qualitative and quantitative traits, respectively. Furthermore, K-mean clustering was executed; the population was grouped into three clusters for both traits. Additionally, the dendrogram using agglomerative hierarchical clustering (AHC), where all the germplasm were grouped into four clusters, revealed that among the clusters, clusters III and IV were highly divergent. The high variability, diversity, and structure could be utilized for the breeding programme of guava and also explored for molecular analysis using next-generation technology to enhance the guava yield and nutrition properties and also develop the climate resilient technology to fulfill the existing demand gap and nutrition availability, which could not only mitigate the nutrition requirement but also enhance the easy availability of fruits year-round.

## Introduction

The lack of diversity in our current eating habits can lead to long-term health issues like allergies, asthma, heart issues, diabetes, cancer, obesity, and also underdevelopment in children and pregnant women in underdeveloped and developing countries of the world. More than 2 billion people are threatened by a global nutritional crisis (1). A healthy diet is crucial for the good health of human beings, but diets deficient in macro and micronutrients are detrimental to those who cannot afford a variety of cuisines (2). The recommended amount of fruit to be consumed per day is 200 g (3). The availability of fruits worldwide reached 248 g per person per day, but different demographic groups consume different amounts of fruits (https://www.fao.org/3/cb9574en/cb9574en.pdf). Contemporary field-crop-centric agricultural practices challenge the nutritional security of the growing population and cause severe health issues. The health-related problems are further aggravated due to changing climatic conditions. Human beings with nutrient deficits are supposed to be more vulnerable to changing climates; the situation will be more alarming if the same trends continue. The impacts of anthropogenic climate change on the climate at local, regional, and global scales are now beyond dispute. It is noted that there have been variations in the frequency and length of extreme climatic occurrences (4). As a result of extreme hot and cold conditions in major cities and metropolitan areas, urban heat islands (UHI) pose a serious threat to human health, agriculture, and forest fires. Climate change and global warming have an impact on biological, physiological, and biochemical processes in fruit crops (5). Specifically, the effect on vegetative development, blooming, fruit set, fruit quality, the occurrence of physiological problems, the prevalence of pests and diseases, shifting cultivars, shifting crops, and other factors, that are eventually exhibiting indicators of decline in fruit production and productivity. Hence, there is a need to exploit the nutrient-rich, climate-resilient fruit crop germplasm that can withstand changing climes.

Guava belongs to the Myrtaceae family, which encompasses 3,300 species and 150 genera (6). By virtue of its hardness and adaptability, guava is extensively dispersed in the tropics. Guava is also known as the “Apple of the Tropics” (7) and it is also well known as “Poor Man's Apple” because of its availability irrespective of the season and a layman can afford it. Guava can be grown in both tropical and subtropical climate conditions (8). Guava has been grown in many countries in the world, but the top guava-producing nations are India, China, Pakistan, Mexico, Brazil, Egypt, Thailand, Columbia, and Indonesia. According to (9), guava was grown in an area of 0.308 million ha in India, with a production of 4.582 million metric tons and a productivity of 14.87 tons/ha. Guava is wellknown for its flavor, delectable taste, and high nutritional value; it contains a lot of vitamins and minerals, *i.e.*, vitamin C or ascorbic acid (100 g of fresh fruit provides 228 mg), vitamin A (140+ g retinol equivalents/100 g), carbohydrates (13%), and other minerals like calcium, phosphorus, and iron, which are also present in the fruit. Guava fruits have strong antioxidant and free radical scavenging properties (10). Many phytochemical compounds are found in the guava fruits, *viz*., saponins, lyxopyranoside, oleanolic acid, arabopyranoside, quercetin, guaijavarin, phenolic compounds, and flavonoids. Guava is a delicate, nutritive, and remunerative fruit crop across the tropical and subtropical regions of the world (11). Guava fruits are easy to process into a variety of nutrient-rich products. Vitamin C is indispensable for growth, development, and tissue repair in the human body (12). Guava is a natural source of vitamin C content that is ~2–5 times higher than that of citrus (13). Furthermore, fruit also possesses an array of medicinal properties (14). Hence there is a need to harness the potential of these wonder fruits by selecting suitable genotypes to cater to the fruit and nutrient requirements of the galloping population. Guava is a self-pollinated crop, but cross-pollination does also occur. The incidence of 35% outcrossing, on the other hand, can provide a heterozygous population with sufficient broader genetic variability to develop attractive commercial varieties (15). Genetic diversity describes the variety of unique inherited traits found in a species (16). Plant breeders have the opportunity to create new and improved cultivars with desirable characteristics that include both farmer-preferred traits (quantity and quality, etc.) and breeder-preferred traits (photosensitivity, pest and disease resistance, etc.). A qualitative technique has been used by (17) for evaluating the fruit diameter of the calyx cavity relative to fruit diameter, skin color, pulp color, and surface texture traits. Similarly, researchers worked on different qualitative traits (18, 19). Based on physical and biochemical characteristics, genetic diversity in the guava and allied *Psidium* species was screened and characterized (20–22). In guava breeding programmes, we must discover underlying genetic diversity across various germplasm/gene pools to find the best parents, which not only enhance yield and fruit quality but also resistance to biotic and abiotic stress environments (23–25).

**Figure 1:**
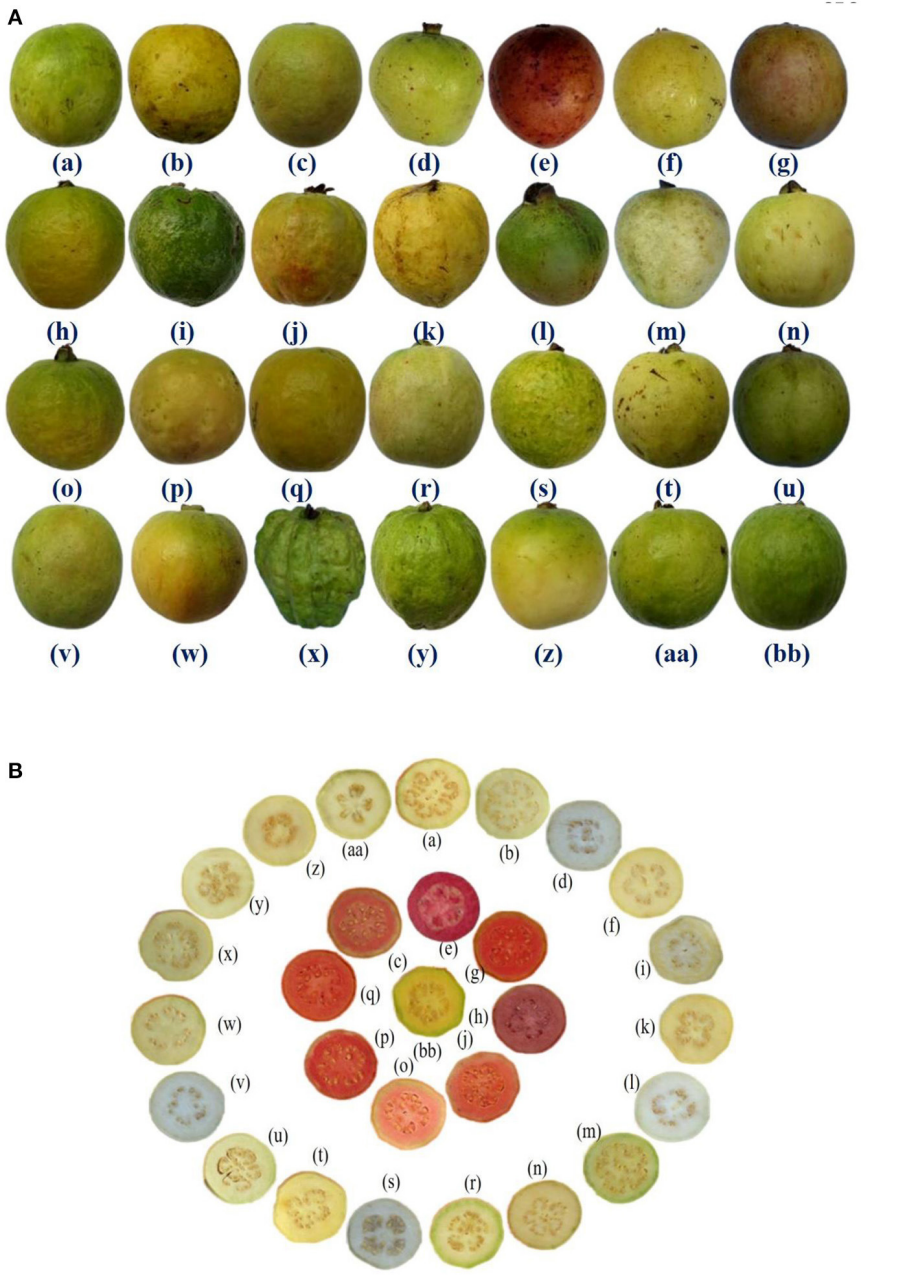
**(A)** Fruit morphology of 28 guava germplasm selected for study. **(B)** Transverse section of fruits of 28 guava germplasm selected for study. (a) Allahabad Safeda, (b) Allahabad Safeda variant, (c) Arka Kiran, (d) Arka Mridula, (e) Black guava (f) Hisar Safeda, (g) Hisar Surkha, (h) Hisar Surkha variant, (i) Kasipur collection, (j) Lalit, (k) Lucknow-49, (l) *P. friedrichsthalianum*, (m) *P. pumilum*, (n) Pant Prabhat, (o) Punjab Pink, (p) Red type I, (q) Red type II, (r) Sasni, (s) Sasri, (t) Shweta, (u) Snow White, (v) Soft Seeded variant, (w) Sour type, (x) Thai, (y) Thai variant, (z) Trichy, (aa) VNR & (bb) Yellow type.

The studied diverse guava germplasm was maintained at the institute farm, which encompasses cultivars, varieties, hybrids, collections, and related species belonging to the genus *Psidium* (see Figures 1A,B). With a wide range of genetic diversity that includes recombination and transgressive segregates, it is possible to obtain allelic variability from individuals in a population that will help to crop for wider adaptability in vagaries environmental conditions. Studied qualitative factors such as fruit size, shape, pulp texture, peel color, pulp color, fruit dots, pulp flavor, *etc*., are the most important for gaining the acceptance of consumers and farmers. Similarly, the quantitative trait which includes fruit weight, length, pulp thickness, pulp weight, ascorbic acid, total soluble solids, antioxidant capacity, *etc*., are crucial for export and processing firms as well as for farmer and consumer acceptance. Quantitative classification offers a divergence value among individuals and thus enables breeders to understand the racial affinities and evolutionary patterns in various species of cultivated plants. It aids in decision-making on the optimal parental combinations to use in a hybridization programme as well. It provides a solid foundation for combining any two or more genotypes depending on their degree of divergence or similarity.

Hence, the present study was objectively conducted to assess the genetic variability and genetic diversity in the selected guava germplasm for the development of elite genotypes. The outcome of the present investigation will help plant breeders to design and develop nutrient-rich high-yielding guava genotypes that can potentially minimize nutritional deficiency and abridge the guava demand and supply gap.

## Materials and methods

### Plant materials

The present investigation was carried out on 28 diverse germplasm of guava (pedigree details are mentioned in Table 1 and Figures 1A,B), and these germplasm were evaluated at the orchard of the Division of Fruits and Horticultural Technology, ICAR-Indian Agricultural Research Institute (IARI), New Delhi during the winter seasons of 2018–19, 2019–20, and 2020–21. The trees were 8 years of age, planted at a spacing of 6 × 3 meters apart, and maintained under ideal horticultural operations.

**Table 1:** Details of 28 guava (*Psidium guajava* L.) germplasm.

**Germplasm name**	**Origin**	**Species name**	**Characteristics**
Allahabad Safeda	Selection	*P. guajava*	White pulp, round fruits
Allahabad Safeda variant	Selection	*P. guajava*	White pulp
Arka Kiran	Hybrid (Kamsari × Purple Local)	*P. guajava*	Pink pulp
Arka Mridula	Selection (Open pollinated seedlings of Allahaba Safeda)	*P. guajava*	White pulp
Black guava	Selection	*P. guajava*	Pink pulp, purple peel
Hisar Safeda	Hybrid (Allahabad Safeda × Seedless)	*P. guajava*	White pulp
Hisar Surkha	Hybrid (Apple color × Banarasi Surkha)	*P. guajava*	Pink pulp
Hisar Surkha variant	Hybrid (Apple color × Banarasi Surkha)	*P. guajava*	Pink pulp, pyriform fruit
Kasipur collection	Selection	*P. guajava*	White pulp
Lalit	Selection (Half sib population of Apple color)	*P. guajava*	Pink pulp, Transversely elliptic fruits
Lucknow-49	Selection (Open pollinated seedlings of Allahabad Safeda)	*P. guajava*	White pulp
*P. friedrichsthalianum*	Related species	*P. friedrichsthalianum*	White pulp, small fruits
*P. pumilum*	Related species	*P. pumilum*	White pulp, small fruits
Pant Prabhat	Selection	*P. guajava*	White pulp
Punjab Pink	Hybrid (Portugal × L-49) × Apple color	*P. guajava*	Pink pulp
Red type I	Selection	*P. guajava*	Pink pulp
Red type II	Selection	*P. guajava*	Pink pulp
Sasni	Selection	*P. guajava*	White pulp
Sasri	Selection	*P. guajava*	White pulp
Shweta	Selection (Half sib population of Apple color)	*P. guajava*	White pulp
Snow White	Selection	*P. guajava*	White pulp
Soft Seeded variant	Selection	*P. guajava*	White pulp, small fruits
Sour type	Selection	*P. guajava*	White pulp, small fruits
Thai	Selection	*P. guajava*	White pulp, large fruits
Thai variant	Selection	*P. guajava*	White pulp, large fruits
Trichy	Selection	*P. guajava*	White pulp, small fruits
VNR	Selection	*P. guajava*	White pulp
Yellow type	Selection	*P. guajava*	Yellow pulp

### Location

The ICAR-IARI, New Delhi is located in the northern region of India (28.4^0^N, 77.1^0^E), with an average rainfall of 797.3 mm per year, 39 mean rainy days, and an average temperature of 34^0^ C.

#### Trait evaluation

A total of 33 qualitative and quantitative traits were recorded in a set of 28 germplasm. The observations are as follows:

### Assessing qualitative traits

In the investigation, a total of 17 fruit attributes (qualitative) such as fruit size (FS), shape (FSH), base shape (FBS), shape at the stalk end (FSS), cavity (FC), pulp texture (FPT), puffiness (FP), calyx persistence (FCP), a diameter of calyx concerning that of fruit (DC), fruit ridged collar around calyx cavity (FRC), surface (FSU), peel color (FPC), pulp color (FPU), absence/presence of fruit dots (FD), fruit surface covered in crimson or cherry red color (FSC), taste (FT), and pulp flavor (FPF) were recorded as per (26).

### Assessing quantitative traits

A total of 16 morpho-biochemical parameters (quantitative) such as fruit weight (FW), fruit length (FL), fruit breadth (FB), fruit index *i.e.*, fruit length/breadth ratio (FI), pulp thickness (PT), seed core cavity (SC), pulp thickness: seed core cavity ratio (PS), pulp weight (PW), pulp percent (PP), pulp: seed weight ratio (PSW), vitamin C or Ascorbic acid (AA), total soluble solids (TSS), titratable acidity (TA), total soluble solids: titratable acidity ratio (TT), antioxidant capacity (AC), and total phenolic content (TP) were recorded in the guava germplasm. For each trait, samples were analyzed in five replicates.

### Methodology for trait measurement

The fruit weight and the pulp weight were recorded using an electronic weighing balance (Aczet CY 223C, India) and expressed in grams. The fruit's length, breadth, pulp thickness, and seed core cavity were measured using Vernier calipers (Mitutoyo 500-754-10, Japan) and expressed in centimeters. A drop of guava juice was placed on the prism of the digital refractometer (MA871 Milwaukee, Romania) to determine TSS and expressed in Brix. Titratable acidity was determined by titrating the aliquot of a known quantity of sample against 0.1N NaOH solution to a pink endpoint using 1% phenolphthalein indicator (27). The recorded titratable acidity was presented as a percent of citric acid. By using a redox titration with potassium iodate and potassium iodide, ascorbic acid was determined (28) and presented in mg/100g of fruit.

The antioxidant capacity in guava fruits was determined by (29). A test tube containing ethanolic fruit extract (0.1 mL) was filled with 1.0 mL each of ammonium acetate buffer, 7.5 × 10^−3^ M neocuproine solution, 10^−2^ M CuCl_2_ solution, and distilled water. After 30 mins, the absorbance of the sample was recorded at 450 nm with a double beam UV-VIS spectrophotometer (UV5704SS, India) against the reagent blank, and the findings were expressed as Trolox equivalent (μmol Trolox/g FW).

Total phenolic content was determined according to the Folin-Ciocalteu reagent (30) using a UVD-3200 spectrophotometer (Labomed, Inc., Culver city, USA). The 80% ethanol was used to smash 2 g of fruit, which was then centrifuged at 10,000 rpm for 20 mins at 4°C. The supernatant was added with 2.5 ml of 0.2 N Folin-Ciocalteu reagents (FCR) and kept for 5 mins, later 2 ml of a 20% sodium carbonate solution was added, and the volume was increased to 25 ml by adding 80% ethanol. The mixture was allowed to set for one and a half hours without disturbance. The absorbance of the mixture was measured at 750 nm, where the intensity of the blue color was relative to the concentration of total phenolic content. The standard calibration curve was developed by using gallic acid. The total phenolic content was expressed as gallic acid equivalent per 100 g extract (mg GAE/100 g extract).

### Statistical analysis

Descriptive statistics including the minimum, maximum, mean, standard deviation, and coefficient of variation were analyzed using Web Agri Stat Package-2 (WASP-2) developed by ICAR Complex Goa, India. Qualitative data was exposed to non-parametric Spearman correlations, whereas quantitative data was submitted to parametric Pearson correlations. Principal component Analysis (PCA) and K-mean cluster plots were used to analyze data. Data sets may be divided into K clusters, which are represented by their centroids, using the K-means clustering technique (31).

A dendrogram was created by using both qualitative and quantitative traits. Based on Ward's approach and Euclidean distance, respectively, aggregative hierarchical clustering (AHC) and genetic dissimilarity component analysis were performed.

The R statistical software (version 4.2.0; The R Foundation) was used to analyze the reported data for correlation, PCA, K-mean cluster plots, and dendrogram of 33 attributes from the 28 guava germplasm.

## Results

Genetic variability and genetic diversity are of prime importance for the selection of the desirable lines, which serve as the basis for designing the breeding programme in the guava crop for improved nutritional status.

### Descriptive statistical analysis and correlations that describe the qualitative traits

Table 2 displays the descriptive statistics of the minimum, maximum, mean, standard deviations, and coefficient of variation (CV) for 17 qualitative traits, which include both morphological and fruit quality traits. The study showed that there was wider and more significant diversity and also had high CV values in the studied traits in the guava germplasm.

**Table 2:** Descriptive statistics for 17 qualitative traits in 28 guava germplasm.

**Trait**	**Maximum**	**Minimum**	**Mean**	**Std. deviation**	**CV**
Fruit size	7.00	3.00	4.64	1.34	28.85
Fruit shape	5.00	1.00	1.89	1.10	58.12
Fruit base shape	2.00	1.00	1.46	0.51	34.68
Fruit shape at the stalk end	5.00	1.00	2.32	0.98	42.36
Fruit cavity	2.00	1.00	1.18	0.39	33.09
Fruit pulp texture	2.00	1.00	1.50	0.51	33.95
Fruit puffiness	9.00	1.00	5.57	4.03	72.36
Fruit calyx persistence	7.00	3.00	4.57	1.26	27.56
Diameter of calyx concerning that of fruit	2.00	1.00	1.57	0.50	32.07
Fruit ridged collar around calyx cavity	7.00	3.00	5.14	1.21	23.50
Fruit surface	2.00	1.00	1.39	0.50	35.71
Fruit peel color	3.00	1.00	1.79	0.57	31.81
Fruit pulp color	3.00	1.00	1.68	0.91	53.91
Fruit dots	2.00	1.00	1.50	0.51	33.95
Fruit surface covered by crimson or cherry red color	7.00	3.00	3.36	0.95	28.33
Fruit taste	7.00	3.00	5.21	1.57	30.15
Fruit pulp flavor	7.00	3.00	5.07	1.39	27.33

The variation of CV values for the traits ranged from 23.5 to 72.36 % in the studied qualitative traits. The higher CV values were observed for the FP (72.36%), FSH (58.12%), and FPC (53.91%). FSS (42.36%), FSU (35.71%), FBS (34.68%), FPT and FD (33.95%), FCP (33.09%), FRC (32.07%), FPC (31.81%), and FT (30.15%) all had medium CV values. Similarly, low CV values included FS (28.85%), FSC (28.33%), DC (27.56%), FPF (27.33%), and FC (23.5%). The variation of CV values showed a higher variation prevalence in the studied germplasm.

According to (26), all germplasm were grouped based on traits such as FS (small- 9, medium- 15, large- 4), FSH (subglobose- 14, ovate- 6, pyriform- 6, oblong- 6, transversely elliptic- 1), FBS (flattened- 15, broadly rounded- 13), FSS (broadly rounded- 6, rounded- 10, truncate- 10, pointed- 1, necked- 1), FC (shallow- 4, medium- 18, deep- 6), FPT (gritty- 14, firm-14), FP (absent- 12, present- 16), FCP (persistent- 23, dropping- 5), DC (small- 9, medium-16, large-3), FRC (inconspicuous- 12, conspicuous- 16), FSU (smooth- 17, warty- 11), FPC (Green-yellowish- 8, yellowish- 17, reddish- 2), FPU (white- 17, creamy white- 3, pinkish- 8), FD (absent- 14, present- 14), FSC (low- 24, intermediate- 3, high- 1), FT (low- 24, intermediate- 3, high- 1) and FPF (low- 6, intermediate- 15, high- 7).

Furthermore, large fruit size was observed in Thai, Thai variant, Allahabad Safeda, and Allahabad Safeda variant. Similarly, the reddish peel color was observed in Black guava and Lalit. Pinkish pulp color was observed in Arka Kiran, Black guava, Hisar Surkha, Hisar Surkha variant, Lalit, Punjab Pink, Red type I, and Red type II, whereas yellow pulp color was seen in the Yellow type germplasm. These descriptions will help to identify and easily trace the germplasm during the breeding programme.

**Figure 2:**
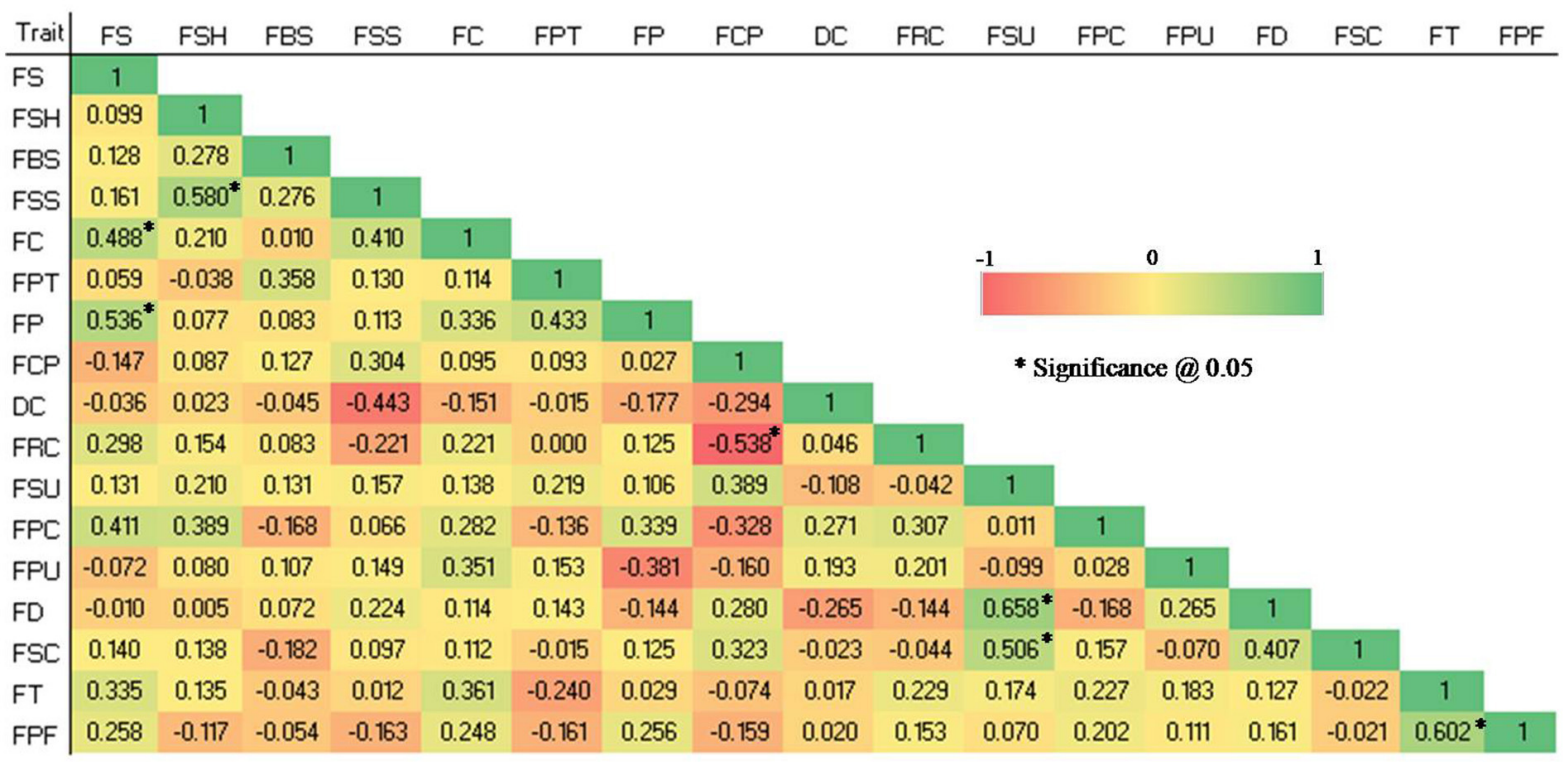
Spearman correlation coefficient among 17 qualitative traits in 28 guava germplasm. FS, fruit size; FSH, fruit shape; FBS, fruit base shape; FSS, fruit shape at stalk end; FC, fruit cavity; FPT, fruit pulp texture; FP, fruit puffiness; FCP, fruit calyx persistence; DC, diameter of calyx concerning that of fruit; FRC, fruit ridged collar around calyx cavity; FSU, fruit surface; FPC, fruit peel color; FPU, fruit pulp color; FD, fruit dots; FSC, fruit surface covered by crimson or cherry red color; FT, fruit taste and FPF, pulp flavor.

Significant correlations (P 0.05) were found in the studied qualitative traits (see Figure 2). The FS and FC (0.488), FS and FP (0.536), FSH and FSS (0.58), FSU and FD (0.658), FSU and FSC (0.506), and FT and FPF (0.602) were shown to have significant positive correlations, whereas FCP and FRC (-0.538) showed a significant negative correlation.

### Principal component analysis (PCA) and K-mean clustering for the qualitative traits

PCA was carried out using 17 qualitative traits; these traits were divided into eight components, which account for 84.2% of the total variation observed in the studied germplasm (Table 3).

**Table 3:** First 8 components from the PCA analysis of 17 qualitative traits in 28 guava germplasm.

**Trait**	**PC1**	**PC2**	**PC3**	**PC4**	**PC5**	**PC6**	**PC7**	**PC8**
Fruit size	0.389	−0.126	0.254	0.013	−0.125	−0.004	0.068	−0.003
Fruit shape	0.242	0.067	−0.246	−0.496	−0.042	−0.230	0.290	−0.029
Fruit base shape	0.075	0.170	0.232	−0.221	0.318	0.262	0.615	−0.061
Fruit shape at the stalk end	0.215	0.313	0.123	−0.227	0.263	−0.426	−0.018	0.016
Fruit cavity	0.408	0.036	0.123	0.089	0.181	−0.193	−0.288	0.200
Fruit pulp texture	0.069	0.212	0.338	−0.192	0.137	0.513	−0.269	0.170
Fruit puffiness	0.277	−0.010	0.484	−0.003	−0.311	0.086	−0.108	0.124
Fruit calyx persistence	−0.030	0.466	0	0.114	−0.151	−0.138	0.154	0.318
Diameter of calyx concerning that of fruit	−0.015	−0.269	−0.277	−0.245	−0.048	0.398	0.139	0.518
Fruit ridged collar around calyx cavity	0.233	−0.310	0.022	−0.134	0.129	0.169	−0.058	−0.611
Fruit surface	0.236	0.362	−0.171	0.080	−0.238	0.320	0.117	−0.188
Fruit peel color	0.338	−0.249	−0.113	−0.250	−0.210	−0.092	−0.091	0.124
Fruit pulp color	0.128	−0.018	−0.283	−0.053	0.605	0.088	−0.297	0.191
Absence/presence of fruit dots	0.164	0.388	−0.265	0.219	0.101	0.215	−0.146	−0.228
Fruit surface covered by crimson or cherry red color	0.213	0.166	−0.382	−0.128	−0.357	0.079	−0.206	−0.040
Fruit taste	0.323	−0.132	−0.162	0.379	0.123	−0.063	0.337	0.094
Fruit pulp flavor	0.268	−0.175	−0.031	0.488	0.056	0.087	0.178	0.132
Variability (%)	18.205	15.860	11.892	9.912	9.556	7.681	5.556	5.366

The first component accounts for 18.2% of the total variation, which encompasses 11 qualitative traits such as FS, FSH, FSS, FC, FP, FRC, FSU, FPC, FSC, FT, and FPF. The second component explained 13.04% of the total variation for the traits (07), *viz.*, FSS, FCP, DC, FRC, FSU, FPC, and FD. The third component explains 11.9% of the total variation, which includes FS, FSH, FPT, FP, DC, FPU, FD, and FSC. The fourth component, the total variation, accounts for 9.9% of the traits like FSH, FPC, FT, and FPF. The fifth component covers 9.6% of the total variation for the traits (FBS, FP, and FSC). The sixth component, explaining 7.7% of the total variation, is comprised of FBS, FSS, FPT, DC and FSU. The seventh component accounts for 5.6% of the total variation for FBS and FT. The eight components explained 5.4% of the total variation for the traits (FCP, DC, and FRC).

**Figure 3:**
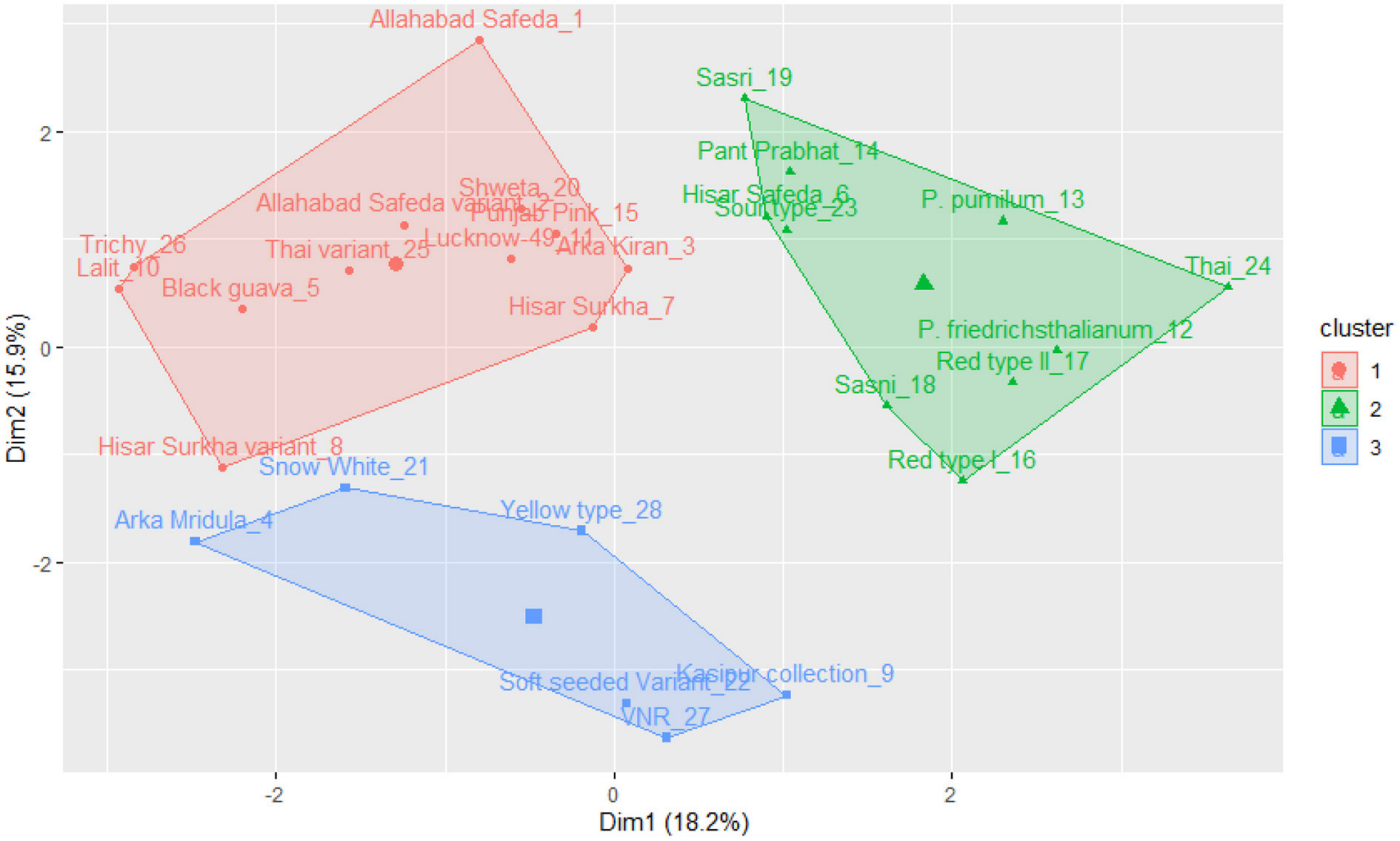
K-means cluster solutions plotted against the PCA scatter plot (component 1 on the X axis and component 2 on the Y axis, k-3) based on the 17 qualitative traits.

The k-means clustering was carried out for 17 qualitative traits of germplasm (a set of 28 germplasm), and this germplasm was grouped into three clusters (see Figure 3). Cluster I encompasses Allahabad Safeda, Lucknow-49, Shweta, Lalit, Punjab Pink, Arka Kiran, Trichy, Black guava, Allahabad Safeda variant, Hisar Surkha variant, and Thai variant. Cluster II comprises Sasri, Pant Prabhat, Hisar Safeda, Sour type, *P. pumilum, P. friedrichsthalianum*, Thai, Red type I, Red type II, and Sasni. Cluster III consists of Arka Mridula, Snow White, Yellow type, Kasipur collection, Soft Seeded variant, and VNR. These results suggested that the K-means clustering approach was efficient to explain the genetic material was widely scattered throughout the plot based on the phenotypic composition and attributes.

### Descriptive statistical analysis and correlation that describe the quantitative traits

In the study, a total of 16 quantitative traits were examined in a set of 28 germplasm lines of guava. The descriptive statistics revealed an enormous amount of variation present among the studied germplasm. The coefficient of variation (CV) ranged from 1.39 to 58.62%. The traits such as PW (58.62%) and FW (58.17%) had high CV values, the trait PSW (33.05%) had a medium CV value, and the rest of the traits showed lower CV values (Table 4).

**Table 4:** Descriptive statistics for 16 quantitative traits in 28 guava germplasm.

**Trait**	**Maximum**	**Minimum**	**Mean**	**Std. deviation**	**CV**
Fruit weight (g)	359.32	17.04	136.39	79.34	58.17
Fruit length (cm)	9.32	2.43	6.08	1.45	23.92
Fruit breadth (cm)	8.82	2.74	6.06	1.41	23.24
Fruit index	1.29	0.82	1.03	0.10	10.19
Pulp weight (g)	336.27	15.37	127.17	74.55	58.62
Pulp percent (%)	93.98	88.34	92.86	1.29	1.39
Pulp thickness (cm)	1.85	0.58	1.27	0.30	23.93
Seed core cavity (cm)	5.40	1.58	3.53	0.94	26.66
Pulp thickness: seed core cavity ratio	1.50	0.50	0.75	0.20	27.05
Pulp: seed weight ratio	91.58	20.16	46.45	15.35	33.05
Ascorbic acid (mg/100 g fruit)	197.27	110.44	147.59	23.29	15.78
Titratable acidity (%)	0.69	0.30	0.44	0.11	25.29
Total soluble solids (^0^B)	15.02	9.49	12.23	1.51	12.34
TSS: titratable acidity ratio	49.35	13.85	30.57	9.17	29.99
Antioxidant capacity (μmol Trolox/g)	44.49	17.53	27.74	7.71	27.79
Total phenolic content (mg GAE/100 g)	186.93	105.63	142.26	19.88	13.97

Similarly, the morpho-biochemical variability was observed among the studied traits in the germplasm lines *i.e*., the maximum FW (359.32 g), FL (9.32 cm), and FB (8.82 cm) in the Thai variety; FI (1.29) and TP (186.93 mg GAE/100 g) in Punjab Pink; PT (1.85 cm) in Allahabad Safeda variant; SC (5.4 cm) in Thai variant; PS (91.58) and TSS (15.02 ^0^B) in Trichy; AA (197.27 mg/100 g fruit) in Lucknow-49; TA (0.69%) in *P. friedrichsthalianum*; TT (49.35) in Allahabad Safeda; and AC (44.49 μmol Trolox/g) in Shweta.

In contrast, the minimum FW (17.04 g), FB (2.43 cm), PW (15.37 g), PT (0.58 cm), SC (1.58 cm), TSS (9.49 ^0^B) and TT (13.85) were recorded in *P. friedrichsthalianum*; FL (2.43 cm), FI (0.82), PP (88.34) and PSW (20.16) in *P. pumilum*; AA (110.44 mg/100g fruit) in the Sour type; AC (17.53 μmol Trolox/g) in Kasipur collection; and TP (105.63 mg GAE/ 100 g) in Yellow type.

**Figure 4:**
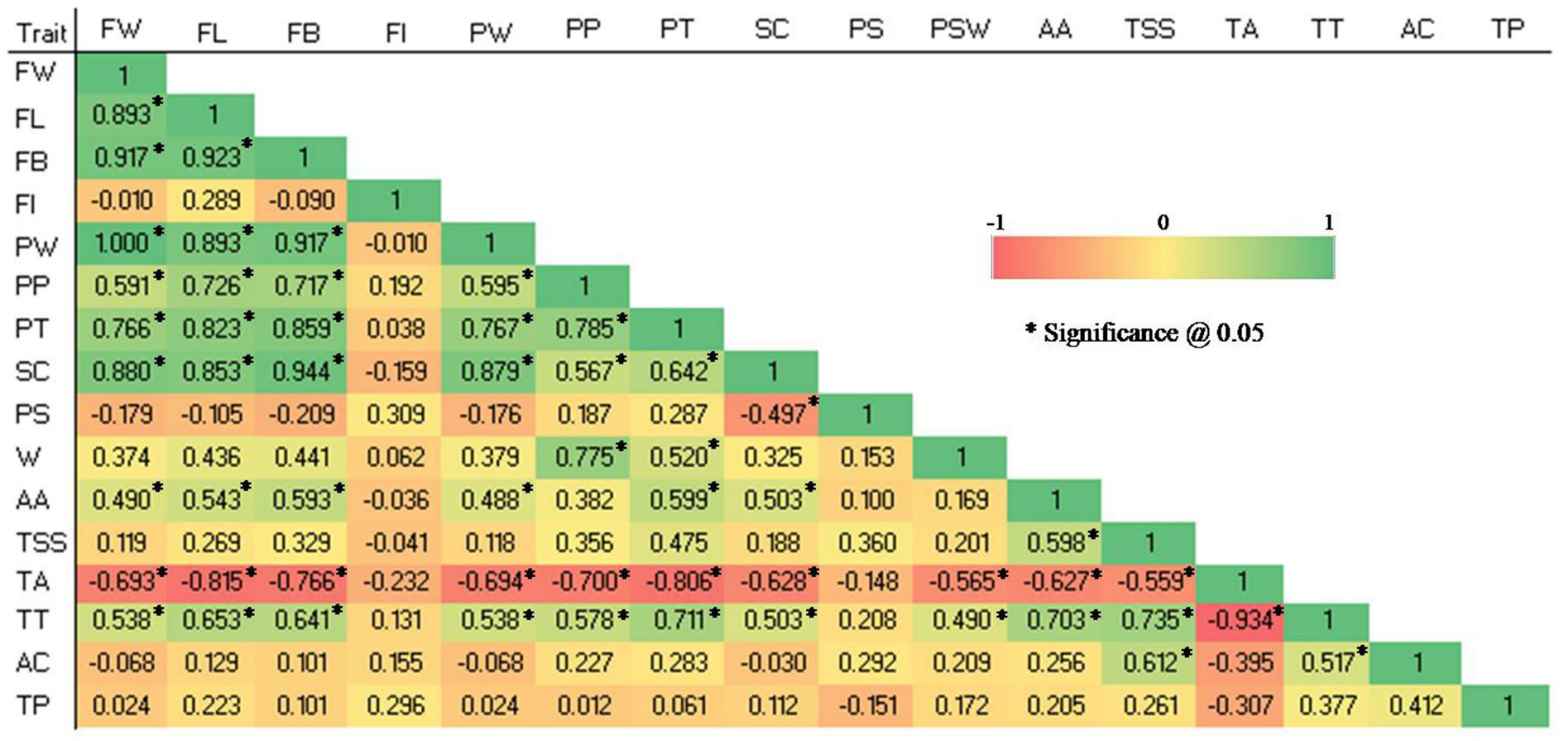
Pearson correlation coefficient among 16 quantitative traits in 28 guava germplasm. FW, fruit weight; FL, fruit length; FB, fruit breadth; FI, fruit index; PT, pulp thickness; SC, seed core cavity; PS, pulp thickness: seed core cavity ratio; PW, pulp weight; PP, pulp percent; PSW, pulp: seed weight ratio; AA, ascorbic acid; TSS, total soluble solids; TA, titratable acidity; TT, TSS, TA ratio; AC, Antioxidant capacity and TP, total phenolic content.

Highly significant positive as well as negative correlations (p 0.05) were observed among studied quantitative traits in the germplasm (see Figure 4).

The following traits showed highly significant positive correlations: FW and PW (1), FB and SC (0.944), FL and FB (0.923), FW and FB (0.917), FB and PW (0.917), FW and FL (0.893), FL and PW (0.893), FW and SC (0.88), PW and SC (0.879), FB and PT (0.859), FL and SC (0.853), FL and PT (0.823), PP and PT (0.785), PP and PSW (0.775), PW and PT (0.767), FW and PT (0.766), TSS and TT (0.735), FL and PP (0.726), FB and PP (0.717), PT and TT (0.711), AA and TT (0.703).

Similarly, significantly positive correlations were reported among the following traits: FW and PP (0.591), FW and AA (0.49), FW and TT (0.538), FL and AA (0.543), FL and TT (0.653), FB and AA (0.593), FB and TT (0.641), PW and PP (0.595), PW and AA (0.488), PW and TT (0.538), PP and SC (0.567), PP and TT (0.578), PT and SC (0.642), PT and PSW (0.520), PT and AA (0.599), SC and AA (0.503), SC and TT (0.503), PSW and TT (0.49), AA and TSS (0.598), TSS and AC (0.612), and TT and AC (0.517).

Significant negative correlations were observed between the following traits: TA and FW (-0.693), TA and FL (-0.815), TA and FB (-0.766), TA and PW (-0.694), TA and PP (-0.7), TA and PT (-0.806), TA and SC (-0.628), TA and PSW (-0.565), TA and AA (-0.627), TA and TSS (-0.559), TA and TT (-0.934), and SC and PS (-0.497).

### Principal component analysis and K-mean clustering for the quantitative traits

In the present investigation, the PCA was performed using 16 quantitative traits. The results revealed that 93.3% of the total variation was observed in the six components of PCA. This shows a large amount of variation exists in the studied germplasm (Table 5).

**Table 5:** First 6 components from the PCA analysis of 16 quantitative traits in 28 guava germplasm.

**Trait**	**PC1**	**PC2**	**PC3**	**PC4**	**PC5**	**PC6**
Fruit weight	0.304	−0.258	−0.045	0.016	−0.140	0.060
Fruit length	0.327	−0.108	−0.052	0.207	−0.161	0.124
Fruit breadth	0.331	−0.178	0.054	−0.055	0.013	0.130
Fruit index	0.032	0.215	−0.304	0.634	−0.425	0.045
Pulp weight	0.304	−0.257	−0.049	0.016	−0.135	0.058
Pulp percent	0.280	0.050	−0.355	0.003	0.278	0.040
Pulp thickness	0.319	0.048	−0.170	−0.164	−0.066	0.127
Seed core cavity	0.290	−0.296	0.191	0.022	0.060	0.114
Pulp thickness: seed core cavity ratio	0.006	0.419	−0.465	−0.238	−0.278	−0.082
Pulp weight: seed weight ratio	0.202	0.095	−0.331	0.088	0.659	−0.331
Ascorbic acid	0.240	0.115	0.270	−0.231	−0.314	−0.471
Titratable acidity	0.174	0.391	0.249	−0.308	−0.024	0.014
Total soluble solids	−0.322	−0.144	0.001	−0.071	0.027	0.081
TSS: titratable acidity ratio	0.292	0.254	0.145	−0.038	−0.010	−0.172
Antioxidant capacity	0.100	0.447	0.209	0.027	0.186	0.696
Total phenolic content	0.080	0.224	0.424	0.552	0.158	−0.263
Variability (%)	50.551	16.89	8.939	8.098	5.792	2.979

The first component, explaining 50.6% of the total variation, was observed in traits such as FW, FL, FB, PW, PP, PT, SC, PSW, AA, TSS, TA, and TT. The second component accounts for 16.9% of the total variation and includes FW, PW, SC, PST, TSS, TT, AC, and TP. The third component, explaining 8.9% of the total variation, is the traits, *viz.*, FI, PP, PST, PSW, AA, TSS, and TP. The fourth component showed an 8.1% total variation (FI and TP). The fifth component covers 5.8% of the total variation, which includes FI and PSW. The sixth component showed 3% of the total variation, and the traits included AA and AC.

**Figure 5:**
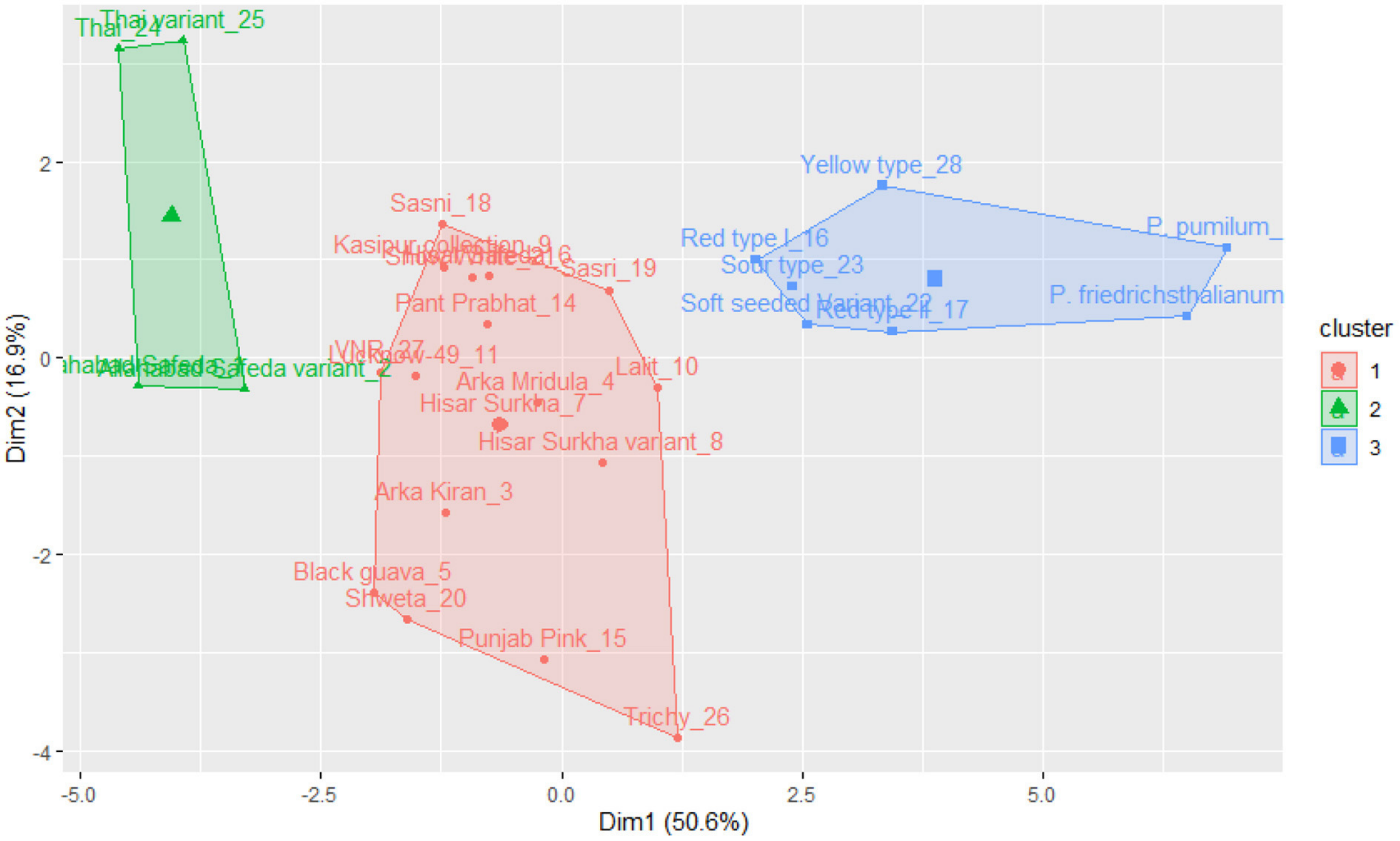
K-means cluster solutions plotted against the PCA scatter plot (component 1 on the X axis and component 2 on the Y axis, k-3) based on the 16 quantitative traits.

The k-means cluster was constructed using the same quantitative traits, and it was grouped into three clusters (see Figure 5). Cluster I includes Thai, Thai variant, Allahabad Safeda, and Allahabad Safeda variant, and this germplasm has shown higher FW, FL, FB, and PW. In contrast, Cluster II contains the germplasm of Yellow type, Red type I, Sour type, Soft seeded variant, Red type II, *P. friedrichsthalianum*, and *P. pumilum*, but these germplasm have lower FW, FL, FB, and PW. The germplasm such as Sasni, Kasipur collection, Hisar Safeda, Sasri, Pant Prabhat, VNR, Lucknow-49, Arka Mridula, Hisar Surkha, Lalit, Hisar Surkha variant, Arka Kiran, Black guava, Shweta, Punjab Pink, and Trichy fall under the cluster III.

### Dendrogram using agglomerative hierarchical clustering (AHC)

**Figure 6:**
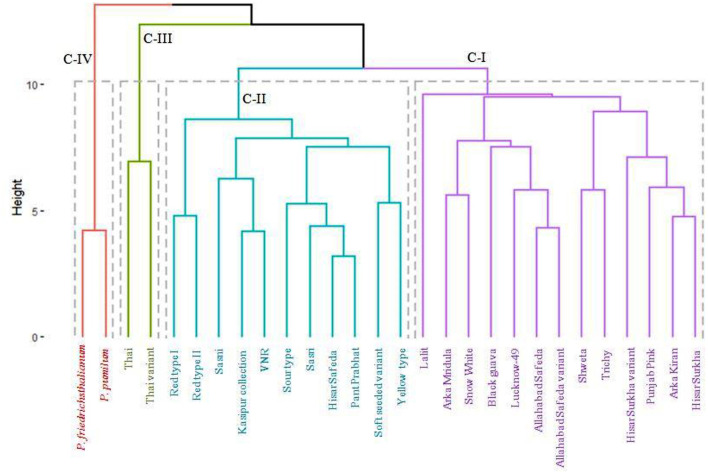
Dendrogram for 28 guava germplasm based on 17 qualitative and 16 quantitative traits.

Based on Euclidian distance and agglomeration using the Wards technique, the genetic dissimilarity of the 28 germplasm samples was performed (see Figure 6). Based on the 17 qualitative and 16 quantitative traits, the dendrogram was divided into four clusters. Cluster-I had thirteen germplasm (Hisar Surkha, Arka Kiran, Punjab Pink, Hisar Surkha variant, Trichy, Shweta, Allahabad Safeda variant, Allahabad Safeda, Lucknow-49, Black guava, Snow White, Arka Mridula, and Lalit).

Cluster II consists of eleven germplasm (Yellow type, Soft seeded variant, Pant Prabhat, Hisar Safeda, Sasri, Sour type, VNR, Kasipur collection, Sasni, Red type II, and Red type I). Cluster III included two germplasm, *i.e.*, Thai and Thai variant, which were the most divergent. On the other hand, related species of guava, *i.e*., *P. pumilum* and *P. friedrichsthalianum* were present in cluster IV. Interestingly, all pink pulp color germplasm falls into clusters I and II.

## Discussion

Guava is an important and emerging fruit crop because of its nutritional properties. Its area is expanding in India and it is an excellent source of raw materials for the processing industry. Ascorbic acid, pectin, phenolic compounds, and antioxidants are abundant in guava, which provides health advantages. Researchers have paid a lot of attention to improving the nutritional quality of most fruit crops such as apples, mangos, bananas, and citrus, but guava has high nutritional values even though they have paid less attention to improvement. Hence, there is immense scope for researchers to improve not only the yield potential but also enhance the nutritional value of guava. To produce novel varieties with farmer and market-preferred product profiles, the genetic diversity found in the examined guava germplasm may be investigated for quantity and quality attributes with modern tools. Therefore, this study was initiated to provide a thorough genetic diversity analysis to reveal the performance and nutritional quality attributes among germplasm collections.

### Morphological traits

In the study, a total of 33 qualitative as well as quantitative traits were studied in a set of 28 guava germplasm to determine the morphological traits that could be useful for genotype identification. It is necessary to characterize and assess germplasm before selecting the required genetic resources for genetic improvement projects. The majority of the traits under investigation had potential economic value, particularly those contributing to fruit yield and fruit quality. These traits can be further targeted for the improvement of guava varieties and hybrids.

The results from the present investigation support the view that morphology and chemical composition in fruits can be utilized efficiently for cultivar discrimination and estimating the genetic relationships in the diverse groups of guava germplasm. These results, which follow those of earlier research, show that both quantitative and qualitative traits are essential for cultivar identification and evaluation in guava germplasm for traits like fruit dots, puffiness, texture, surface, cavity, pulp taste, etc., which are of interest to taxonomists or breeders (17, 32). Similarly, quality traits are the most important factor in attracting consumers and farmers. In the current research, a significant variance was observed in traits such as fruit length/breadth ratio, fruit breadth, fruit length, fruit size, fruit diameter, fruit ridges on skin, fruit length of the stalk, fruit juiciness, total soluble solid, fruit relief of the surface, fruit ridges, the thickness of outer flesh, fruit skin color, fruit shape at the stalk, longitudinal ridges, flesh color, and fruit acidity (33, 34). Similarly, variation was observed for fruit weight, surface, shape, rind color, pulp color, pulp texture, pulp flavor, taste, and TSS (18, 35, 36).

From the perspective of farmers, consumers, export purposes, and processing industries, quantitative traits such as fruit weight, length, breadth, pulp weight, pulp thickness, pulp percent, pulp: seed core cavity ratio, pulp: seed weight ratio, TSS, antioxidants, and so on are some of the most important.

(22) observed higher vitamin C in red and white guavas than in strawberry guava. Red guavas are suited for processing due to their pulp color, higher beta-carotene, phytochemicals, and minerals. Similarly, (21) reported that the genotypes of cluster IV (Allahabad Safeda, Nagpur Seedless, Parkers Dessert, Kohir Safeda, Lalit, Lucknow-49, and Nagpur Seedless) and cluster V (Lucknow-49, VNR-Bihi selection line) are highly heterozygous, and these lines are valuable for breeding programmes.

### Morphological correlations

The degree of correlation between the traits is a crucial consideration, particularly for complicated and economically important traits like yield (37). The ranking values for each variable are used as the basis for the Spearman correlation coefficient rather than the raw data. In the current research, highly positive correlations were observed among qualitative traits (fruit size with cavity and puffiness; fruit size with dots; and fruit taste with flavor). Similarly, some of the qualitative traits showed a significantly negative correlation, *i.e.*, calyx persistence and fruit ridged collar around the calyx cavity. Positive correlation ensures simultaneous improvement in two or more variables, and negative correlation brings out the need to obtain a compromise between the desirable characteristics (34).

The linear relationship between two continuous variables is assessed using the Pearson correlation (38). A strong and significant correlation was found between quantitative traits, particularly fruit weight, length, breadth, pulp weight, pulp thickness, and vitamin C. Based on the significant positive correlations between the fruit yield and quality traits such as fruit weight, fruit breadth, fruit length, cavity diameter, longitudinal ridge, and longitudinal ridge prominence, it can be understood that these characters positively influence the assessment of cultivar potential. The results are in agreement with previous works on guava fruit characters (34, 39, 40), and biochemical parameters (8, 41).

### Principal component analysis (PCA)

PCA is used to uncover patterns and remove duplication in data sets since differences in crop species for yield and quality occur often (42). The main advantage of PCA is that it quantifies the importance of each dimension for correlating a dataset's variability. In the study, a wide range of total variations was observed, *i.e*., 84.2 and 93.3% for qualitative and quantitative traits, respectively. The primary two components from the PCA demonstrate the highest loading for some traits, including fruit size, shape, cavity, puffiness, peel color, taste, pulp flavor, fruit weight, fruit length, fruit breadth, pulp weight, pulp percent, pulp thickness, seed core cavity, pulp: seed cavity weight, vitamin C, total soluble solids, etc., (43, 44). This outcome helps with the diversity assessment and also with the characterization of guava germplasm.

### K-mean clustering for the qualitative and quantitative traits

K-mean clustering was used in this study to group 28 guava germplasm lines into three clusters for qualitative and quantitative traits.

One cluster of varieties exhibited similar traits and had less diversity variation. Cluster analysis makes it possible to identify groups of germplasm that share a variety of traits, which may be helpful for picking the best parent for a crossing. Crossing between varieties from the same group or closely related groups may result in less variation, whereas crossing between groups that are far apart will result in more variation. These lines could be attempted in genetic improvement programmes, either for obtaining transgressive segregants or hybrids (45).

### Dendrogram using agglomerative hierarchical clustering (AHC)

In the present investigation, four clusters were formed among 28 guava germplasm based on the dissimilarity concerning 17 qualitative and 16 quantitative traits. For further advancements, plant breeding is mostly dependent on the genetic variability of cultivated and wild relatives. The pink color of guava pulp is due to the naturally occurring organic pigment carotenoid. All of the pink-colored germplasm is present in clusters I and II, which may be explained by the qualitative and quantitative characteristic values falling within the acceptable range. Among the clusters, cluster III (Thai and Thai variant) and cluster IV (*P. pumilum* and *P. friedrichsthalianum*) were the most divergent for studied traits, hence the selection of such germplasm is important for effective utilize in crop improvement programmes in guava. Similar results were observed by (8), where three distinct clusters based on the dissimilarities of eight indigenous guava cultivars were seen.

## Conclusions

Overall, the results revealed the presence of significant genetic variation among the 28 guava germplasm for both qualitative and quantitative traits, which provides an opportunity for the selection of superior genotypes for breeding programmes as parents in hybridization for nutritionally rich variety development. Among the studied germplasm, Thai, Thai variant, Trichy, Lucknow-49, Allahabad Safeda, and Shweta showed promising for some traits. These germplasm can be explored in a breeding programme to enhance the yield as well as nutritional quality.

Even though morphological traits are typically used to infer genetic links, such assessments are better with constraints. Unfortunately, the environment and a plant's stage of development have a big impact on these morphometric properties, which may lead to an unanticipated variety of agronomically useful characteristics. Future marker-assisted breeding and next generation sequence analysis could be used for guava crop improvement may benefit from a combinational approach to identifying and associating DNA-based molecular markers targeting promising fruit characteristic loci. Species that have great genetic diversity are especially capable of overcoming difficulties when new pests, illnesses, and climatic changes develop.

## Data availability statement

The original contributions presented in the study are included in the article/supplementary material, further inquiries can be directed to the corresponding author/s.

## Author contributions

NG, CA, MV, AN, and MT contributed to the organization of the work plan for the manuscript. NG, AN, and CS drafted the manuscript and composed the outline. VY, MV, KR, and MT contributed to the data analysis. AS, RS, and MS wrote the sections of the manuscript. All the authors had full access to the data and revised and approved the manuscript for publication.

## Funding

This work was funded by the Post Graduate School, ICAR-Indian Agricultural Research Institute, New Delhi.

## Conflict of interest

The authors declare that the research was conducted in the absence of any commercial or financial relationships that could be construed as a potential conflict of interest.

## Publisher's note

All claims expressed in this article are solely those of the authors and do not necessarily represent those of their affiliated organizations, or those of the publisher, the editors and the reviewers. Any product that may be evaluated in this article, or claim that may be made by its manufacturer, is not guaranteed or endorsed by the publisher.
